# Dietary Inflammatory Index and Risk of Colorectal Cancer: A Case-Control Study in Korea

**DOI:** 10.3390/nu8080469

**Published:** 2016-07-30

**Authors:** Young Ae Cho, Jeonghee Lee, Jae Hwan Oh, Aesun Shin, Jeongseon Kim

**Affiliations:** 1 Molecular Epidemiology Branch, National Cancer Center, Goyang 10408, Korea; youngcho914@gmail.com (Y.A.C.); jeonghee@ncc.re.kr (J.L.); 2 Center for Colorectal Cancer, National Cancer Center Hospital, National Cancer Center, Goyang 10408, Korea; jayoh@ncc.re.kr; 3 Department of Preventive Medicine, Seoul National University College of Medicine, Seoul 03080, Korea

**Keywords:** colorectal cancer, inflammation, diet, interaction

## Abstract

The role of diet-associated inflammation in colorectal cancer is of interest. Accordingly, we aimed to examine whether the dietary inflammatory index (DII) was associated with the risk of colorectal cancer in a case-control study conducted in Korea. The DII was based on dietary intake, which was determined by a 106-item semi-quantitative food frequency questionnaire completed by 923 colorectal cancer cases and 1846 controls. Logistic regression was used to estimate odd ratios (ORs) and 95% confidence intervals (CIs). Subgroup analyses were conducted by the anatomical site of the cancer, sex, and other risk factors. Higher DII scores were associated with an increased incidence of colorectal cancer (OR (95% CI) = 2.16 (1.71, 2.73) for highest vs. lowest tertile). The magnitude differed by anatomical site and sex. This association was slightly weaker in subjects with proximal colon cancer (1.68 (1.08, 2.61)) and was stronger in women (2.50 (1.64, 3.82)). Additionally, stronger associations were observed in subjects who were older than 50 years (*p* for interaction = 0.004) and engaged in physical activity (*p* for interaction < 0.001). Results from this study suggest that diet-associated inflammation may increase the risk of colorectal cancer, and this effect may differ by certain factors, such as anatomical site, age, sex, and lifestyle.

## 1. Introduction

Chronic inflammation is known to play an important role in cancer, particularly in colorectal cancer [1]. Evidence suggests that chronic inflammatory bowel disease increases the risk of colorectal cancer development [2,3] and that the long-term use of aspirin and other non-steroidal anti-inflammatory agents is associated with a reduced risk of colorectal cancer [4]. Several studies have indicated that specific components of the diet may modulate inflammation. For example, fruit, vegetable, fiber, and moderate alcohol intake may decrease inflammation, whereas red meat, processed meat, and fat may increase inflammation [5,6,7]. However, the analysis of individual foods or nutrients does not allow for consideration of the complicated interactions or high inter-correlations between dietary factors, and the effect of any single nutrient may be too small to detect [8]. Combining dietary factors, such as in dietary patterns, may overcome these limitations [8], but this methodology may still have difficulty explaining the underlying mechanisms and applying them to diverse populations.

Recently, the dietary inflammatory index (DII) was developed based on an extensive literature review to evaluate the inflammatory potential of an individual’s diet [9,10]. The DII was designed with a focus on inflammation. Additionally, it standardizes individual dietary intake to world reference values and can, thus, be used in diverse populations worldwide [9,10]. Since human diets include both pro-inflammatory and anti-inflammatory components, the DII score may more accurately reflect the relationship between diet and colorectal cancer risk than individual dietary components or other diet indices [11]. The DII has been found to be associated with inflammatory cytokines, including C-reactive protein and IL-6 [12,13,14]. An association between higher DII scores and colorectal cancer has been observed in several studies conducted in Italy [9] and in the US [15,16,17], suggesting that pro-inflammatory diets play a role in colorectal carcinogenesis.

The incidence of colorectal cancer in Korea has increased rapidly in recent decades [18]. In addition to other factors, unhealthy dietary habits have been suggested to elevate the risk of colorectal cancer. Therefore, we aimed to examine the association between the DII and the risk of colorectal cancer through a case-control study conducted in Korea.

## 2. Materials and Methods

### 2.1. Study Population

The cases were patients newly diagnosed with colorectal cancer between August 2010 and August 2013 at the Center for Colorectal Cancer of the National Cancer Center in Korea. Of the 1070 patients who agreed to participate in the study and provided informed consent, 145 were excluded because of incomplete semi-quantitative food frequency questionnaire (SQFFQ) data, and two were removed because of implausible energy intakes (<500 kcal/day or >4000 kcal/day). Therefore, 923 patients were included in the analysis.

The controls were selected from subjects who visited the Center for Cancer Prevention and Detection of the same hospital for a health check-up provided by the National Health Insurance Cooperation, which covers the entire Korean population, between October 2007 and December 2014. Of the 14,201 subjects who agreed to participate in the study, 5044 were excluded because of missing data on the FFQ (*n* = 4779) and other questionnaires (*n* = 265) and 120 subjects were excluded because of implausible energy intakes. Of the remaining 9037 subjects, two controls per case were frequency-matched by age (within five years) and sex (Figure S1).

All participants provided written informed consent and the study protocol was approved by the Institutional Review Board of the National Cancer Center (IRB No. NCCNCS-10-350 and NCC2015-0202).

### 2.2. Dietary Assessment and Calculation of the DII

Information on demographic and lifestyle risk factors of both cases and controls were collected using a structured questionnaire conducted at the initial recruitment, prior to cancer diagnosis. The dietary intake of each participant was assessed using a 106-item SQFFQ. The validity and reproducibility of the questionnaire have been previously reported [19]. Participants provided their individual average frequency of eating and typical portion sizes in the year preceding the interview. These values were converted to obtain daily nutrient intake using a scale with the nine frequency categories (never or rarely, once a month, twice or thrice a month, once or twice a week, three or four times a week, five or six times a week, once a day, twice a day, and three times a day) and three portion size categories (small, medium, and large) included in the SQFFQ. The SQFFQ-derived data were used to calculate the DII scores of all participants. The details of the development [10] and construct validation [12,20] of the DII have been previously described. To calculate the DII score, we used the method previously reported by Shivappa et al. [10]. Specifically, this study included the following 36 food items to generate the DII score: protein, fat, carbohydrate, fiber, monounsaturated fatty acid, saturated fatty acid, polyunsaturated fatty acid, *n*-3 fatty acid, *n*-6 fatty acid, cholesterol, thiamin, riboflavin, niacin, vitamin B_6_, vitamin B_12_, vitamin C, folic acid, vitamin A, vitamin D, vitamin E, β-carotene, iron, magnesium, selenium, zinc, ethanol, garlic, ginger, onion, green tea, flavan-3-ols, flavones, flavonols, flavanones, anthocyanidins, and isoflavones. Energy adjustment was performed using the residual method [21].

### 2.3. Data Analysis

The differences between cases and controls were analyzed according to demographic and lifestyle factors using chi-square tests for categorical variables and *t*-tests for continuous variables. The scores on the DII were categorized into tertiles according to the distribution of the control groups (for all subjects: T1: <0.30, T2: 0.30 to 2.30, T3: ≥2.30; for men: T1: <0.66, T2: 0.66 to 2.51, T3: ≥2.51; for women: T1: <−0.28, T2: −0.28 to 1.76, T3: ≥1.76). The differences in demographic and lifestyle factors and the distributions of the intake of specific food groups (cereals and grain products, potatoes and starches, sugars, legumes, seeds and nuts, vegetables, mushrooms, fruits, meat, eggs, fish and shellfish, seaweed, milk and dairy products, fats and oils, beverages, seasoning) were examined across the DII tertiles. ANOVA was used to test for differences.

Odds ratios (ORs) and 95% confidence intervals (CIs) were estimated using logistic regression for both the crude and multivariable models. The multivariable model was adjusted for age, sex, body mass index, education, family history of colorectal cancer, physical activity, and total caloric intake. A linear test for trend was conducted by including the median value of each DII tertile as a continuous term in the regression model. For the subgroup analyses by anatomical location, polytomous logistic regression model was used. The outcome variables included colorectal cancer overall and subsites (colon, proximal colon cancer, distal colon cancer, and rectal cancer). The anatomical subsites were defined based on the International Statistical Classification of Disease and Related Health Problems, 10th revision (ICD-10) [22].

Potential effect modification of the association between the DII and colorectal cancer risk was examined by stratifying the data by age group (<50 years old and ≥50 years old), BMI (<25 kg/m^2^ and ≥25 kg/m^2^), physical activity (yes/no), and smoking status (ever/never). The interactions between the DII scores and risk factors were assessed with a likelihood ratio test comparing the model with interaction terms with the model containing only the main effects.

All statistical analyses were performed using SAS^®^ 9.2 software (SAS Institute Inc., Cary, NC, USA). A two-sided *p*-value less than 0.05 was considered statistically significant.

## 3. Results

The distribution of participant characteristics is presented in Table 1 for controls and cases. There were some significant differences between cases and controls in terms of socio-demographic factors and lifestyle habits: cases were more likely to have a family history of colorectal cancer (*p* < 0.001), were less highly educated (*p* < 0.001), were less likely to have professional occupations (*p* < 0.001), had lower incomes (*p* < 0.001), were less likely to be married (*p* < 0.001), were less likely to drink alcohol (*p* < 0.001), and had lower physical activity (*p* < 0.001) than controls. The total calorie intake was 1689.9 kcal/day among controls and 2026.3 kcal/day among cases (*p* < 0.001). The mean DII value was 1.07 and 1.80 for controls and cases (*p* < 0.001), respectively, indicating a more pro-inflammatory diet among the cases. The DII score in the present study ranged from a maximum pro-inflammatory score of 7.26 to a maximum anti-inflammatory score of −6.50, with a standard deviation of 2.18.

Some differences in demographic and lifestyle factors across tertiles of the DII were observed in controls (Table S1). Those with a higher DII score were more likely to be men (*p* < 0.001), were more likely to perform physical labor (*p* < 0.001), had lower incomes (*p* = 0.008), and had lower physical activity (*p* < 0.001) than those with a lower DII score. The distribution of certain food groups across tertiles of the DII was also examined in both controls and cases (Table S2). Concerning the distribution of various food groups, cereals and grain products showed a significant increase across the tertiles of the DII, but the other food groups, except for sugars and fats and oils, evinced a significant decrease across the tertiles of the DII. The controls and cases showed different distributions of sugars, meats, and fats and oils.

Table 2 shows the association between the DII score and colorectal cancer risk by anatomical location. A higher DII score (representing a more pro-inflammatory diet) was associated with an increased incidence of colorectal cancer (OR (95% CI) = 2.16 (1.71, 2.73) for highest vs. lowest tertile, *p* for trend < 0.001). When the data were stratified by anatomic site, a slightly weaker association was observed with proximal colon cancer (OR (95% CI) = 1.68 (1.08, 2.61) for highest vs. lowest tertile, *p* for trend = 0.02) (Table 2). When the data were stratified by sex, a stronger association was observed among women (OR (95% CI) = 2.50 (1.64, 3.82) for highest vs. lowest tertile, *p* for trend < 0.001) compared with men (OR (95% CI) = 1.72 (1.30, 2.28) for highest vs. lowest tertile, *p* for trend < 0.001) (Table 3).

The potential effect modification of the association between the DII and colorectal cancer by age, BMI, physical activity, and smoking status was investigated (Table 4). Analysis of the strata of potential effect modifiers showed differences in the associations between DII and colorectal cancer by age group (*p* for interaction = 0.004), physical activity (*p* for interaction < 0.001), and smoking status (*p* for interaction = 0.03). Stronger associations were observed among subjects who were 50 years and older (OR (95% CI) = 2.61(1.98, 3.43) for highest vs. lowest tertile), engaged in physical activity (OR (95% CI) = 3.42 (2.37, 4.95) for highest vs. lowest tertile) and did not smoke (OR (95% CI) = 2.58 (1.81, 3.68) for highest vs. lowest tertile).

## 4. Discussion

This study found that a more pro-inflammatory diet was associated with an increased risk of colorectal cancer. The magnitude of the risk varied slightly by anatomic location and sex. In addition, the association between colorectal cancer risk and diet-associated inflammation may be modified by factors such as age and physical activity.

The DII was developed to assess the inflammatory potential of an entire diet by integrating individual food components [10]. This method could compensate for the limitations of previous methods and can, thus, be used without concern regarding dietary intercorrelations and the particular dietary culture of the study population [8,9,10,15]. However, some limitations should be considered in the interpretation of the study findings. First, this study was a case-control study and, thus, selection and recall biases may have affected the results. The controls were those who visited a health screening center and, therefore, they may have been more health conscious than the general population; additionally the cases and controls may have differed in their recall of dietary habits. However, the data were gathered using a validated questionnaire without knowledge of the specific hypotheses of this study. In addition, the cases were newly diagnosed colorectal cancer patients, and the assessment of dietary intake was conducted before diagnoses of cancer, thereby reducing the potential for differential misclassification and measurement errors [23]. Second, only dietary habits over the previous year were assessed in this analysis. Although the effects of long-term dietary habits on inflammatory potential were not incorporated, adult dietary habits tend to remain stable [24]. Third, residual or unmeasured confounding due to non-dietary factors (e.g., non-steroidal anti-inflammatory drugs, hormone replacement therapy, or other drug use) may have affected the results.

The results of this study were similar to those of previous studies using the DII. Two US cohort studies of postmenopausal women, the Iowa Women’s Health Study [15] and the Women’s Health Initiative [17], reported that the highest quintile of the DII was associated with a 20% increased risk of colorectal cancer. Another US cohort study of 489,422 men and women 50–74 years old reported a 40% increase in the risk of colorectal cancer among those in the highest quartile of DII scores compared with the lowest quartile [16]. Two case-control studies conducted in Italy [9] and Spain [25] reported similar findings. In addition, previous studies using single foods/nutrients [5,6,7] or dietary indices [26,27,28,29] support this finding. The dietary components affecting colorectal cancer risk are generally consistent with their inflammatory potential [10]. Numerous studies have reported that the consumption of red and processed meat, fiber, whole grains, and folate is associated with colorectal carcinogenesis [5,6,7]. Additionally, dietary patterns high in fruits, vegetables, fish, poultry, and whole grains are associated with a lower colorectal cancer risk, while those high in red or processed meat, refined grains and sweets are associated with an increased colorectal cancer risk [26,27,28,29].

Several mechanisms have been proposed to explain the effect of diet-related inflammation in colorectal carcinogenesis. First, systemic inflammation is responsible for increasing insulin resistance [30] which may affect colorectal cancer risk via the growth-promoting effects of elevated insulin, glucose, or triglycerides [31,32]. In addition, activation of the COX-2 pathway may result in focal proliferation, angiogenesis, and mutagenesis [2]. COX-2 can be upregulated by inflammatory stimuli via inflammatory cytokines (e.g., IL-6) and growth factors, and it can be downregulated by dietary components (e.g., vitamin D and *n*-3 fatty acids), physical activity, and medications [33]. Moreover, other dietary component may affect carcinogenesis. Diets high in red and processed meats (which are more pro-inflammatory) may be high in *N*-nitroso compounds, which can damage DNA [34]. Diets high in fruits and vegetables (which are more anti-inflammatory) contain antioxidants and micronutrients, which have anti-tumor capabilities, and fiber, which can reduce the transit time of food in the digestive tract [34].

We observed slightly different effects of diet-associated inflammation by anatomical site. In this study, relatively smaller effects were observed in subjects with proximal colon cancer. Colorectal cancer is a heterogeneous disease, presenting different clinical and molecular characteristics by anatomic location [35,36]. For example, receptors for insulin and insulin-like growth factors and pH levels are reported to differ in proximal and distal locations [33,36]. These variations may influence susceptibility to inflammation by anatomic location [36]. Additionally, we found slightly stronger effects among women. The mechanisms by which environmental factors influence risk of colon and rectal cancer are suggested to be more complex and less consistent in women than in men because of the possible influence of the actions of estrogen [37,38].

Other factors may affect the role played by diet-associated inflammation in colorectal carcinogenesis. In this study, stronger associations were observed among subjects who were older than 50 years and those who engaged in physical activity. The incidence of colorectal cancer increases sharply after the age of 50 [39], suggesting a stronger effect of environmental risk factors, including diet, rather than genetic factors [18]. Physical activity is related to both inflammation and colorectal cancer risk [40], and frequent physical activity is associated with lower systemic inflammation and improved insulin sensitivity [13,40]. In the present study, physical inactivity increased the risk of colorectal cancer, but the effect of diet-associated inflammation may be greater among those engaged in physical activity. Similarly, a previous study found a greater effect of an inflammatory marker (IL-6) on colorectal cancer only among lean people [41]. In addition, in this population, different patterns of consumption of certain food groups (e.g., sugars, fats and oils, meat) between cases and controls were observed across DII levels. These findings point to the possibility of more effective diet and lifestyle interventions to prevent colorectal cancer. Further studies of the potential mechanisms driving the differential associations between diet-associated inflammation and colorectal cancer risk are needed.

In conclusion, this study found that consuming a diet with greater pro-inflammatory potential was associated with an increased risk of colorectal cancer in a Korean population, suggesting a role of diet-associated inflammation in colorectal carcinogenesis. This study also suggested that the role of diet-associated inflammation may be affected by several factors, such as anatomical location, age, sex, and lifestyle. These findings may lead to more specific strategies to prevent colorectal cancer in the Korean population that consider both dietary habits and lifestyles. Combined with regular exercise, changing dietary habits by increasing fruit and vegetable consumption, and reducing meat, sweets, and fried foods may help prevent colorectal cancer. The use of this new tool for assessing dietary inflammatory potential could be extended to other inflammation-related diseases.

## Figures and Tables

**Table 1:** General characteristics of the study subjects ^1^.

	Controls (*n* = 1846)	Cases (*n* = 923)	*p*-Value
Age (years)	56.1 ± 9.1	56.6 ± 9.7	0.20
FeMale	596 (32.3)	298 (32.3)	>0.99
Family history of colorectal cancer	99 (5.4)	99 (10.7)	<0.001
BMI (kg/m^2^)			
<25	1226 (66.4)	640 (69.3)	0.12
≥25	620 (33.6)	283 (30.7)	
Educational level			
Middle school or less	282 (15.6)	321 (34.8)	<0.001
High school	587 (32.6)	369 (40.0)	
College or more	934 (51.8)	233 (25.2)	
Occupation			
Professional, office	481 (26.4)	189 (20.5)	<0.001
Service, sales	403 (22.1)	38 (4.1)	
Agriculture, mining, manufacturing	241 (13.2)	141 (15.3)	
Housewife, other	698 (38.3)	555 (60.1)	
Monthly Household Income ^2^			
<200	388 (23.0)	321 (34.8)	<0.001
200−<400	754 (44.7)	387 (41.9)	
≥400	545 (32.3)	215 (23.3)	
Marital Status			
Married	1654 (90.4)	773 (83.8)	<0.001
Unmarried	176 (9.6)	150 (16.3)	
Smoking status			
Non-smoker	818 (44.3)	409 (44.3)	0.16
Former smoker	687 (37.2)	318 (34.5)	
Current smoker	341 (18.5)	196 (21.2)	
Alcohol Consumption			
Non-drinker	560 (30.3)	279 (30.2)	<0.001
Former drinker	169 (9.2)	129(14.0)	
Current drinker	1117 (60.5)	515 (55.8)	
Total caloric intake (Kcal/day)	1689.6 ± 560.4	2026.3 ± 534.0	<0.001
Sum of DII	1.07 ± 2.24	1.80 ± 1.97	<0.001
Physical activity (yes)	1047 (58.2)	311 (33.7)	<0.001

BMI, body mass index; DII, dietary inflammatory index; ^1^ Data are presented as *n* (%) or mean ± SD; ^2^ Unit is 10,000 won in Korean currency (1 $ = 1,168.3 Korean won as of 17 June 2016).

**Table 2:** Associations between dietary inflammatory index and the risk of colorectal cancer, stratified by anatomical site ^1^.

DII Score	No. Controls/Cases	Crude OR (95% CI)	Adjusted ^2^ OR (95% CI)
**Colorectal Cancer**			
T1	616/197	1.0 (ref.)	1.0 (ref.)
T2	615/319	1.62 (1.32, 2.00)	1.99 (1.57, 2.53)
T3	615/407	2.07 (1.69, 2.54)	2.16 (1.71, 2.73)
*p* for trend		<0.001	<0.001
**Colon Cancer**			
T1	616/102	1.0 (ref.)	1.0 (ref.)
T2	615/168	1.65 (1.26, 2.16)	2.08 (1.55, 2.79)
T3	615/190	1.87 (1.43, 2.43)	2.05 (1.53, 2.74)
*p* for trend		<0.001	<0.001
**Proximal Colon Cancer**			
T1	616/38	1.0 (ref.)	1.0 (ref.)
T2	615/66	1.74 (1.15, 2.63)	2.11 (1.37, 3.27)
T3	615/61	1.61 (1.06, 2.45)	1.68 (1.08, 2.61)
*p* for trend		0.02	0.02
**Distal Colon Cancer**			
T1	616/64	1.0 (ref.)	1.0 (ref.)
T2	615/102	1.60 (1.15, 2.23)	2.06 (1.44, 2.93)
T3	615/129	2.02 (1.47, 2.78)	2.28 (1.61, 3.21)
*p* for trend		<0.001	<0.001
**Rectal Cancer**			
T1	616/92	1.0 (ref.)	1.0 (ref.)
T2	615/147	1.60 (1.21, 2.13)	1.90 (1.40, 2.59)
T3	615/205	2.23 (1.70, 2.92)	2.23 (1.66, 3.00)
*p* for trend		<0.001	<0.001

CI, confidence interval; DII, dietary inflammatory index; OR, odds ratio; ref., reference; T, tertile; ^1^ The DII scores were categorized into tertiles according to the distribution of the control groups: for all subjects (T1: <0.30, T2: 0.30 to 2.30, T3: ≥2.30); ^2^ Adjusted for age, sex, BMI, education, family history of colorectal cancer, physical activity, and total calorie intake.

**Table 3:** Associations between dietary inflammatory index and the risk of colorectal cancer, stratified by sex ^1^.

DII Score	Men			Women		
	No. Controls/Cases	Crude OR (95% CI)	Adjusted OR ^2^ (95% CI)	No. Controls/Cases	Crude OR (95% CI)	Adjusted OR ^2^ (95% CI)
**Colorectal Cancer**						
T1	417/150	1.0 (ref.)	1.0 (ref.)	199/56	1.0 (ref.)	1.0 (ref.)
T2	417/211	1.41 (1.10, 1.81)	1.68 (1.26, 2.24)	199/98	1.75 (1.19, 2.57)	1.92 (1.24, 2.98) 3.57)
T3	416/264	1.80 (1.39, 2.25)	1.72 (1.30, 2.28)	198/144	2.58 (1.79, 3.73)	2.50 (1.64, 3.82)
*p* for trend		<0.001	<0.001		<0.001	<0.001
**Colon Cancer**						
T1	417/73	1.0 (ref.)	1.0 (ref.)	199/31	1.0 (ref.)	1.0 (ref.)
T2	417/100	1.37 (0.98, 1.91)	1.70 (1.18, 2.44)	199/58	1.87 (1.16, 3.02)	2.07 (1.22, 3.51)
T3	416/120	1.65 (1.20, 2.27)	1.67 (1.18, 2.37)	199/78	2.53 (1.60, 4.01)	2.52 (1.51, 4.21)
*p* for trend		0.002	0.004		<0.001	<0.001
**Proximal Colon Cancer**						
T1	417/27	1.0 (ref.)	1.0 (ref.)	199/11	1.0 (ref.)	1.0 (ref.)
T2	417/45	1.67 (1.02, 2.74)	2.08 (1.23, 3.50)	199/16	1.46 (0.66, 3.21)	1.48 (0.64, 3.41)
T3	416/40	1.49 (0.90, 2.47)	1.51 (0.89, 2.57)	198/26	2.38 (1.14, 4.94)	2.23 (1.02, 4.89)
*p* for trend		0.11	0.10		0.02	0.04
**Distal Colon Cancer**						
T1	417/46	1.0 (ref.)	1.0 (ref.)	199/20	1.0 (ref.)	1.0 (ref.)
T2	417/55	1.20 (0.79, 1.81)	1.48 (0.96, 2.30)	199/42	2.10 (1.19, 3.70)	2.42 (1.31, 4.48)
T3	416/80	1.74 (1.18, 2.57)	1.76 (1.17, 2.66)	198/52	2.61 (1.51, 4.54)	2.69 (1.47, 4.92)
*p* for trend		0.005	0.007		<0.001	0.002
**Rectal Cancer**						
T1	417/76	1.0 (ref.)	1.0 (ref.)	199/23	1.0 (ref.)	1.0 (ref.)
T2	417/106	1.40 (1.01, 1.93)	1.61 (1.12, 2.31)	199/40	1.74 (1.00, 3.01)	1.85 (1.02, 3.36)
T3	416/138	1.82 (1.33, 2.48)	1.73 (1.23, 2.44)	198/61	2.67 (1.59, 4.48)	2.44 (1.38, 4.32)
*p* for trend		<0.001	0.002		<0.001	0.002

CI, confidence interval; DII, dietary inflammatory index; OR, odds ratio; ref., reference; T, tertile; ^1^ The DII scores were categorized into tertiles according to the distribution of the control groups; for men (T1: <0.66, T2: 0.66 to 2.51, T3: ≥2.51); for women (T1: <−0.28, T2: −0.28 to 1.76, T3: ≥1.76); ^2^ Adjusted for age, BMI, education, family history of colorectal cancer, physical activity, and total calorie intake.

**Table 4:** Associations between dietary inflammatory index and the risk of colorectal cancer, stratified by risk factors ^1^.

	Dietary Inflammatory Index						*p* for Interaction
	T1		T2		T3		
	No. Controls/Cases	OR (95% CI) ^2^	No. Controls/Cases	OR (95% CI) ^2^	No. Controls/Cases	OR (95% CI) ^2^	
**Age**							
<50 years old	116/55	1.0 (ref.)	135/57	0.94 (0.58, 1.55)	169/98	1.17 (0.75, 1.85)	0.004
≥50 years old	500/142	1.0 (ref.)	480/262	2.48 (1.87, 3.28)	446/309	2.61 (1.98, 3.43)	
**BMI (kg/m^2^)**							
<25	399/126	1.0 (ref.)	421/231	2.23 (1.65, 3.00)	406/283	2.34 (1.75, 3.14)	0.58
≥25	217/71	1.0 (ref.)	194/88	1.65 (1.09, 2.49)	209/124	1.84 (1.25, 2.72)	
**Physical Activity**							
No	195/134	1.0 (ref.)	254/211	1.54 (1.12, 2.13)	304/267	1.49 (1.09, 2.03)	<0.001
Yes	411/63	1.0 (ref.)	341/108	2.67 (1.83, 3.89)	295/140	3.42 (2.37, 4.95)	
**Smoking Status**							
Never	310/91	1.0 (ref.)	270/156	2.63 (1.85, 3.75)	239/162	2.58 (1.81, 3.68)	0.03
Ever	307/106	1.0 (ref.)	345/163	1.50 (1.07, 2.09)	376/245	1.79 (1.31, 2.45)	

BMI, body mass index; CI, confidence interval; OR, odds ratio; ref., reference; T, tertile; ^1^ The DII scores were categorized into tertiles according to the distribution of the control groups (T1: <0.30, T2: 0.30 to 2.30, T3: ≥2.30); ^2^ Adjusted for age, sex, BMI, education, family history of colorectal cancer, physical activity, and total calorie intake, if applicable.

## Supplementary Materials

The following are available online at http://www.mdpi.com/2072-6643/8/8/469/s1, Figure S1: Flow diagram of the selection of cases and controls, Table S1: General characteristics of the study subjects according to tertiles of dietary inflammatory index, Table S2: Distribution of food group intake in study participants according to tertiles of dietary inflammatory index.

## Abbreviations

The following abbreviations are used in this manuscript:

BMI	Body mass index
CI	Confidence interval
DII	Dietary inflammatory index
FFQ	Food frequency questionnaire
OR	Odds ratio
SD	Standard deviation
T	Tertile

## References

[1] Coussens L.M., Werb Z. (2002). Inflammation and cancer. Nature.

[2] Triantafillidis J.K., Nasioulas G., Kosmidis P.A. (2009). Colorectal cancer and inflammatory bowel disease: Epidemiology, risk factors, mechanisms of carcinogenesis and prevention strategies. Anticancer Res..

[3] Bernstein C.N., Blanchard J.F., Kliewer E., Wajda A. (2001). Cancer risk in patients with inflammatory bowel disease: A population-based study. Cancer.

[4] Flossmann E., Rothwell P.M. (2007). Effect of aspirin on long-term risk of colorectal cancer: Consistent evidence from randomised and observational studies. Lancet.

[5] Miller P.E., Lazarus P., Lesko S.M., Cross A.J., Sinha R., Laio J., Zhu J., Harper G., Muscat J.E., Hartman T.J. (2013). Meat-related compounds and colorectal cancer risk by anatomical subsite. Nutr. Cancer.

[6] Parr C.L., Hjartaker A., Lund E., Veierod M.B. (2013). Meat intake, cooking methods and risk of proximal colon, distal colon and rectal cancer: The Norwegian Women and Cancer (NOWAC) cohort study. Int. J. Cancer.

[7] Song M., Garrett W.S., Chan A.T. (2015). Nutrients, foods, and colorectal cancer prevention. Gastroenterology.

[8] Hu F.B. (2002). Dietary pattern analysis: A new direction in nutritional epidemiology. Curr. Opin. Lipidol..

[9] Shivappa N., Zucchetto A., Montella M., Serraino D., Steck S.E., La Vecchia C., Hebert J.R. (2015). Inflammatory potential of diet and risk of colorectal cancer: A case-control study from Italy. Br. J. Nutr..

[10] Shivappa N., Steck S.E., Hurley T.G., Hussey J.R., Hebert J.R. (2014). Designing and developing a literature-derived, population-based dietary inflammatory index. Public Health Nutr..

[11] Shivappa N., Jackson M.D., Bennett F., Hebert J.R. (2015). Increased Dietary Inflammatory Index (DII) is associated with increased risk of prostate cancer in Jamaican men. Nutr. Cancer.

[12] Shivappa N., Steck S.E., Hurley T.G., Hussey J.R., Ma Y., Ockene I.S., Tabung F., Hebert J.R. (2014). A population-based dietary inflammatory index predicts levels of C-reactive protein in the Seasonal Variation of Blood Cholesterol Study (SEASONS). Public Health Nutr..

[13] Wirth M.D., Burch J., Shivappa N., Violanti J.M., Burchfiel C.M., Fekedulegn D., Andrew M.E., Hartley T.A., Miller D.B., Mnatsakanova A. (2014). Association of a dietary inflammatory index with inflammatory indices and metabolic syndrome among police officers. J. Occup. Environ. Med..

[14] Wood L.G., Shivappa N., Berthon B.S., Gibson P.G., Hebert J.R. (2015). Dietary inflammatory index is related to asthma risk, lung function and systemic inflammation in asthma. Clin. Exp. Allergy.

[15] Shivappa N., Prizment A.E., Blair C.K., Jacobs D.R., Steck S.E., Hebert J.R. (2014). Dietary inflammatory index and risk of colorectal cancer in the Iowa Women’s Health Study. Cancer Epidemiol. Biomark. Prev..

[16] Wirth M.D., Shivappa N., Steck S.E., Hurley T.G., Hebert J.R. (2015). The dietary inflammatory index is associated with colorectal cancer in the National Institutes of Health-American Association of Retired Persons Diet and Health Study. Br. J. Nutr..

[17] Tabung F.K., Steck S.E., Ma Y., Liese A.D., Zhang J., Caan B., Hou L., Johnson K.C., Mossavar-Rahmani Y., Shivappa N. (2015). The association between dietary inflammatory index and risk of colorectal cancer among postmenopaUSAl women: Results from the Women’s Health Initiative. Cancer Causes Control.

[18] Shin A., Kim K.Z., Jung K.W., Park S., Won Y.J., Kim J., Kim D.Y., Oh J.H. (2012). Increasing trend of colorectal cancer incidence in Korea, 1999–2009. Cancer Res. Treat..

[19] Ahn Y., Kwon E., Shim J.E., Park M.K., Joo Y., Kimm K., Park C., Kim D.H. (2007). Validation and reproducibility of food frequency questionnaire for Korean genome epidemiologic study. Eur. J. Clin. Nutr..

[20] Tabung F.K., Steck S.E., Zhang J., Ma Y., Liese A.D., Agalliu I., Hingle M., Hou L., Hurley T.G., Jiao L. (2015). Construct validation of the dietary inflammatory index among postmenopaUSAl women. Ann. Epidemiol..

[21] Willett W.C. (2012). Nutritional Epidemiology.

[22] World Health Organization (2011). International Statistical Classification of Diseases and Related Health Problems.

[23] D’Avanzo B., La Vecchia C., Katsouyanni K., Negri E., Trichopoulos D. (1997). An assessment, and reproducibility of food frequency data provided by hospital controls. Eur. J. Cancer Prev..

[24] Sijtsma F.P., Meyer K.A., Steffen L.M., Shikany J.M., Van Horn L., Harnack L., Kromhout D., Jacobs D.R. (2012). Longitudinal trends in diet and effects of sex, race, and education on dietary quality score change: The Coronary Artery Risk Development in Young Adults study. Am. J. Clin. Nutr..

[25] Zamora-Ros R., Shivappa N., Steck S.E., Canzian F., Landi S., Alonso M.H., Hébert J.R., Moreno V. (2015). Dietary inflammatory index and inflammatory gene interactions in relation to colorectal cancer risk in the Bellvitge colorectal cancer case-control study. Genes Nutr..

[26] Magalhaes B., Peleteiro B., Lunet N. (2012). Dietary patterns and colorectal cancer: Systematic review and meta-analysis. Eur. J. Cancer Prev..

[27] Miller P.E., Lesko S.M., Muscat J.E., Lazarus P., Hartman T.J. (2010). Dietary patterns and colorectal adenoma and cancer risk: A review of the epidemiological evidence. Nutr. Cancer.

[28] Kim M.K., Sasaki S., Otani T., Tsugane S. (2005). Dietary patterns and subsequent colorectal cancer risk by subsite: A prospective cohort study. Int. J. Cancer.

[29] Chen Z., Wang P.P., Woodrow J., Zhu Y., Roebothan B., McLaughlin J.R., Parfrey P.S. (2015). Dietary patterns and colorectal cancer: Results from a Canadian population-based study. Nutr. J..

[30] Festa A., D’Agostino R., Howard G., Mykkanen L., Tracy R.P., Haffner S.M. (2000). Chronic subclinical inflammation as part of the insulin resistance syndrome: The Insulin Resistance Atherosclerosis Study (IRAS). Circulation.

[31] Bruce W.R., Giacca A., Medline A. (2000). Possible mechanisms relating diet and risk of colon cancer. Cancer Epidemiol. Biomark. Prev..

[32] Giovannucci E. (1995). Insulin and colon cancer. Cancer Causes Control.

[33] Chan A.T., Giovannucci E.L. (2010). Primary prevention of colorectal cancer. Gastroenterology.

[34] Vargas A.J., Thompson P.A. (2012). Diet and nutrient factors in colorectal cancer risk. Nutr. Clin. Pract..

[35] Wei E.K., Giovannucci E., Wu K., Rosner B., Fuchs C.S., Willett W.C., Colditz G.A. (2004). ComParison of risk factors for colon and rectal cancer. Int. J. Cancer.

[36] Iacopetta B. (2002). Are there two sides to colorectal cancer?. Int. J. Cancer.

[37] Terry P.D., Miller A.B., Rohan T.E. (2002). Obesity and colorectal cancer risk in women. Gut.

[38] Chlebowski R.T., Wactawski-Wende J., Ritenbaugh C., Hubbell F.A., Ascensao J., Rodabough R.J., Rosenberg C.A., Taylor V.M., Harris R., Chen C. (2004). Estrogen plus progestin and colorectal cancer in postmenopaUSAl women. N. Engl. J. Med..

[39] US Preventive Services Task Force (2008). Screening for colorectal cancer: US preventive services task force recommendation statement. Ann. Intern. Med..

[40] Pischon T., Hankinson S.E., Hotamisligil G.S., Rifai N., Rimm E.B. (2003). Leisure-time physical activity and reduced plasma levels of obesity-related inflammatory markers. Obes. Res..

[41] Song M., Wu K., Ogino S., Fuchs C.S., Giovannucci E.L., Chan A.T. (2013). A prospective study of plasma inflammatory markers and risk of colorectal cancer in men. Br. J. Cancer.

