# Supplementary material for: Dietary Inflammatory Index and Risk of Colorectal Cancer: A Case-Control Study in Korea
Source: Nutrients. 2016 Jul 30;8(8):469. doi: 10.3390/nu8080469 (PMC4997382; doi:10.3390/nu8080469)
Supplement: Supplementary file 1 [file nutrients-08-00469-s001.docx]

Supplementary Materials: Dietary Inflammatory Index and Risk of Colorectal Cancer: A Case-Control Study in Korea

Young Ae Cho, Jeonghee Lee, Jae Hwan Oh, Aesun Shin and Jeongseon Kim


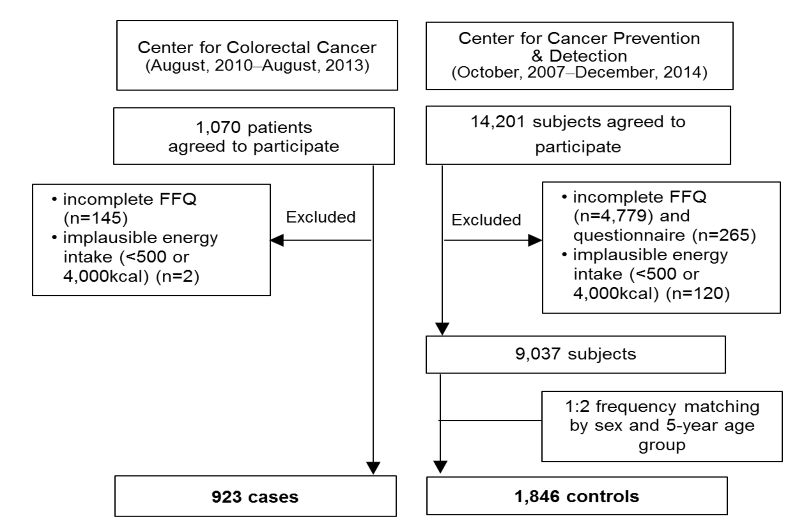


**Figure S1.** Flow diagram of the selection of cases and controls.

**Table S1.** General characteristics according to tertiels of dietary inflammatory index in controls ^1^.

|  | **Dietary Inflammatory Index** | | | ***p*-Value ^2^** |
| --- | --- | --- | --- | --- |
|  | **T1** | **T2** | **T3** |  |
| Age (years) | 56.6 ± 8.6 | 56.4 ± 9.3 | 55.3 ± 9.4 | <0.001 |
| Female | 252 (40.9) | 205 (33.3) | 139 (22.6) | <0.001 |
| Family history of colorectal cancer | 37 (6.0) | 32 (5.2) | 30 (4.9) | 0.67 |
| BMI (kg/m^2^) |  | | | |
| <25 | 399 (64.8) | 421 (68.5) | 406 (66.0) | 0.38 |
| ≥25 | 217 (35.2) | 194 (31.5) | 209 (34.0) |  |
| Educational Level |  | | | |
| Middle school or less | 93 (15.4) | 81 (13.6) | 108 (18.0) | 0.23 |
| High school | 193 (31.9) | 209 (35.0) | 185 (30.8) |  |
| College or more | 319 (51.7) | 307 (51.4) | 308 (51.3) |  |
| Occupation |  | | | |
| Professional, office | 160 (26.2) | 167 (27.7) | 154 (25.3) | <0.001 |
| Service, sales | 135 (22.1) | 115 (19.0) | 153 (25.2) |  |
| Agriculture, mining, manufacturing | 58 (9.5) | 79 (13.1) | 104 (17.1) |  |
| Housewife, others | 258 (42.2) | 243 (40.2) | 197 (32.4) |  |
| Monthly Household Income ^3^ |  | | | |
| <200 | 110 (19.7) | 151 (26.8) | 127 (22.5) | 0.008 |
| 200−<400 | 244 (43.7) | 238 (42.3) | 272 (48.1) |  |
| >400 | 205 (36.7) | 174 (30.9) | 166 (29.4) |  |
| Marital Status |  | | | |
| Married | 561 (92.1) | 540 (88.4) | 553 (90.7) | 0.08 |
| Unmarried | 48 (7.9) | 71 (11.6) | 57 (9.3) |  |
| Smoking Status |  | | | |
| Nonsmoker | 309 (50.2) | 270 (43.9) | 239 (38.9) | <0.001 |
| Former smoker | 213 (34.6) | 219 (35.6) | 255 (41.5) |  |
| Current smoker | 94 (15.3) | 126 (20.5) | 121 (19.7) |  |
| Alcohol Consumption |  | | | |
| Nondrinker | 200 (32.5) | 189 (30.7) | 171 (27.8) | 0.03 |
| Former drinker | 40 (6.5) | 58 (9.4) | 71 (11.5) |  |
| Current drinker | 376 (61.0) | 568 (59.8) | 373 (60.7) |  |
| Total caloric intake (Kcal/day) | 1837.7 ± 551.9 | 1556.3 ± 565.7 | 1674.57 ± 527.8 | <0.001 |
| Physical activity (yes) | 411 (67.8) | 341 (57.3) | 295 (49.3) | <0.001 |

^1^ Data are presented as *n* (%) or mean ± SD; ^2^ *p*-values were obtained from ANOVA; ^3^ Unit is 10,000 won in Korean currency (1 $ = 1168.3 Korean won as of 17 June 2016).

**Table S2.** Distribution of food group intakes according to tertiles of dietary inflammatory index ^1^.

| **Food Groups (g/Day)** | **Controls** | | | | **Cases** | | | |
| --- | --- | --- | --- | --- | --- | --- | --- | --- |
|  | **Dietary Inflammatory Index** | | | ***p*-Value ^2^** | **Dietary Inflammatory Index** | | | ***p*-Value ^2^** |
|  | **T1** | **T2** | **T3** |  | **T1** | **T2** | **T3** |  |
| Cereals/grain product | 547.5 ± 208.7 | 563.4 ± 213.2 | 693.5 ± 196.4 | <0.001 | 747.3 ± 224.9 | 789.6 ± 221.2 | 877.3 ± 200.2 | <0.001 |
| Potatoes/starches | 60.6 ± 48.4 | 36.1 ± 36.2 | 26.0 ± 23.5 | <0.001 | 57.3 ± 39.8 | 44.7 ± 30.2 | 33.1 ± 26.7 | <0.001 |
| Sugars | 5.5 ± 5.8 | 5.1 ± 5.2 | 5.7 ± 6.1 | 0.18 | 7.0 ± 6.8 | 7.3 ± 6.8 | 8.9 ± 7.5 | <0.001 |
| Legume | 82.3 ± 72.4 | 55.3 ± 69.7 | 44.2 ± 58.2 | <0.001 | 75.2 ± 56.9 | 68.1 ± 55.9 | 52.6 ± 72.8 | <0.001 |
| Seeds/nuts | 8.1 ± 16.2 | 4.8 ± 11.9 | 3.1 ± 5.4 | <0.001 | 4.7 ± 8.5 | 3.7 ± 6.6 | 2.1 ± 3.5 | <0.001 |
| Vegetables | 471.9 ± 221.9 | 265.9 ± 127.8 | 183.1 ± 90.5 | <0.001 | 453.0 ± 203.3 | 339.3 ± 151.7 | 237.2 ± 94.5 | <0.001 |
| Mushrooms | 14.0 ± 22.1 | 6.0 ± 7.7 | 4.7 ± 5.5 | <0.001 | 13.4 ± 14.1 | 11.3 ± 9.7 | 8.1 ± 7.2 | <0.001 |
| Fruits | 317.1 ± 302.4 | 152.2 ± 155.8 | 93.6 ± 104.9 | <0.001 | 303.3 ± 243.3 | 177.0 ± 151.3 | 109.1 ± 89.7 | <0.001 |
| Meat | 66.6 ± 52.0 | 54.5 ± 49.72 | 54.1 ± 53.2 | <0.001 | 66.3 ± 46.9 | 63.5 ± 52.3 | 68.1 ± 57.3 | 0.51 |
| Eggs | 20.7 ± 20.9 | 15.3 ± 15.4 | 14.2 ± 14.4 | <0.001 | 19.3 ± 14.7 | 17.4 ± 16.5 | 15.2 ± 16.4 | 0.01 |
| Fish/shellfish | 56.6 ± 44.2 | 34.8 ± 27.2 | 26.0 ± 21.4 | <0.001 | 61.6 ± 39.8 | 50.7 ± 33.0 | 36.2 ± 21.0 | <0.001 |
| Seaweeds | 3.1 ± 2.7 | 1.8 ± 1.9 | 1.2 ± 1.3 | <0.001 | 2.6 ± 1.7 | 2.1 ± 1.4 | 1.4 ± 1.0 | <0.001 |
| Milk/dairy products | 120.1 ± 134.7 | 96.3 ± 118.1 | 88.9 ± 153.0 | <0.001 | 108.3 ± 120.3 | 80.8 ± 90.8 | 76.7 ± 131.2 | 0.006 |
| Fats/oils | 4.1 ± 3.6 | 3.7 ± 3.9 | 4.0 ± 4.1 | 0.21 | 5.3 ± 4.4 | 5.5 ± 5.2 | 6.5 ± 5.5 | 0.005 |
| Beverages | 102.0 ± 127.4 | 71.4 ± 95.1 | 52.5 ± 79.9 | <0.001 | 95.2 ± 117.0 | 68.5 ± 81.5 | 48.6 ± 75.9 | <0.001 |
| Seasoning | 25.2 ± 16.6 | 14.9 ± 12.4 | 10.9 ± 7.0 | <0.001 | 24.4 ± 20.6 | 17.0 ± 10.0 | 12.3 ± 7.3 | <0.001 |

^1^ Data are presented as mean ± SD; ^2^ *p*-values were obtained from ANOVA.
